# Distinct Zika Virus Lineage in Salvador, Bahia, Brazil

**DOI:** 10.3201/eid2210.160663

**Published:** 2016-10

**Authors:** Samia N. Naccache, Julien Thézé, Silvia I. Sardi, Sneha Somasekar, Alexander L. Greninger, Antonio C. Bandeira, Gubio S. Campos, Laura B. Tauro, Nuno R. Faria, Oliver G. Pybus, Charles Y. Chiu

**Affiliations:** University of California San Francisco, San Francisco, California, USA (S.N. Naccache, S. Somasekar, C.Y. Chiu);; University of California San Francisco–Abbott Viral Diagnostics and Discovery Center, San Francisco (S.N. Naccache, S. Somasekar, C.Y. Chiu);; University of Oxford, Oxford, UK (J. Thézé, N.R. Faria, O.G. Pybus);; Federal University of Bahia, Salvador, Brazil (S.I. Sardi, G.S. Campos);; University of Washington, Seattle, Washington, USA (A.L. Greninger);; Hospital Alianca, Salvador (A.C. Bandeira);; Gonçalo Moniz Research Center–Oswaldo Cruz Foundation, Salvador (L.B. Tauro);; Evandro Chagas Institute, Ananindeua, Brazil (N.R. Faria)

**Keywords:** Zika virus, ZIKV, flaviviruses, Bahia, Brazil, mosquito-borne infections, outbreak surveillance, metagenomic next-generation sequencing, viral genome assembly, capture probe enrichment, phylogenetic analysis, molecular clock, viruses, vector-borne infections

## Abstract

Sequencing of isolates from patients in Bahia, Brazil, where most Zika virus cases in Brazil have been reported, resulted in 11 whole and partial Zika virus genomes. Phylogenetic analyses revealed a well-supported Bahia-specific Zika virus lineage, which indicates sustained Zika virus circulation in Salvador, Bahia’s capital city, since mid-2014.

Zika virus is an arthropodborne RNA virus primarily transmitted by mosquitoes of the species *Aedes* (*1*). The virus has 2 genotypes: African, found only in the continent of Africa; and Asian, associated with outbreaks in Southeast Asia, several Pacific islands, and, recently, the Americas (*2*). In May 2015, Brazil reported its first autochthonous cases of Zika virus infection, which occurred in northeast Brazil (*3*,*4*). As of June 30, 2016, all 27 federal states in Brazil had confirmed Zika virus transmission (http://www.paho.org/hq/index.php?option=com_docman&task=doc_view&Itemid=270&gid=35262&lang=en).

The rapid geographic expansion of Zika virus transmission and the virus’s association with microcephaly and congenital abnormalities (*5*) demand a rapid increase in molecular surveillance in areas that are most affected. Molecular surveillance is particularly relevant for regions where other mosquitoborne viruses, particularly dengue and chikungunya viruses, co-circulate with Zika virus (*2*); surveillance on the basis of clinical symptoms alone is highly inaccurate. Genetic characterization of circulating Zika virus strains can help determine the origin and potential spread of infection in travelers returning from Zika virus–endemic countries. Previous analyses have suggested that Zika virus was introduced in the Americas at least 1 year before the virus’s initial detection in Brazil (*1*). The state of Bahia, Brazil, reported most (93%) suspected Zika virus infections in Brazil during 2015 (*2*), including cases of Zika virus–associated fetal microcephaly (*6*); however, except for 1 complete genome, no genetic information from the region has been available (*2*,*7*). We report molecular epidemiologic findings resulting from 11 new complete and partial Zika virus genomes recovered from serum samples from patients at the Hospital Aliança in the city of Salvador in Bahia, Brazil.

## The Study

Symptomatic patients with suspected Zika virus infection were enrolled in a research study approved by the Brazil Ministry of Health (Certificado de Apresentação para Apreciação Ética 45483115.0.0000.0046, no. 1159.184, Brazil). During April 2015–January 2016, acute Zika virus infection was diagnosed for 15 patients whose serum samples tested positive by a qualitative reverse transcription PCR (RT-PCR) by using primers targeting the nonstructural 5 gene (*8*). Clinical samples were retested for Zika virus positivity by using a separate quantitative RT-PCR (QuantiTect SYBR Green PCR kit; QIAGEN, Valencia, CA, USA) and primers targeting the envelope gene (*9*). Metagenomic next-generation sequencing libraries were constructed from serum RNA extracts, as described (*10*,*11*; Technical Appendix). Pathogen identification from metagenomic next-generation sequencing data was performed by using the Sequence-based Ultra-Rapid Pathogen Identification bioinformatics pipeline (*12*; http://chiulab.ucsf.edu/surpi/). Results of the metagenomic analyses and identification of co-infections with chikungunya virus are reported elsewhere (*13*). 

For Zika virus genome sequencing, 2 isolates (Bahia07 and Bahia09; Table) with Zika virus titers >10^4^ copies/mL generated sufficient viral metagenomic data for complete genome assembly. For the remaining samples with lower titers, metagenomic next-generation sequencing libraries were enriched for Zika virus sequencing by using xGen biotinylated lockdown capture probes (Integrated DNA Technologies, Redwood, CA, USA) designed to tile across all sequenced Zika virus genomes >10,000 nt in GenBank (http://www.ncbi.nlm.nih.gov/genbank) as of March 1, 2016. Capture probes were curated for redundancy at a 99% nt similarity cutoff. Enrichment was performed on the metagenomic libraries in pools of 8 libraries (including Zika virus–negative serum sample controls) by using the xGen lockdown probe protocol and the SeqCap EZ Hybridization and Wash Kit (Roche, Indianapolis, IN, USA). Eleven Zika virus genomes with >40% genome recovery (mean 69.4% ± 2.0%) were assembled (Table). Distribution of single nucleotide variants across the 11 recovered genomes exhibited distinct patterns (Technical Appendix Figure 1), indicating that the assembled genomes were unlikely to result from cross-contamination by a single high-titer Zika virus sample.

**Table:** Clinical information for isolates from serum samples of patients with acute symptomatic Zika virus infection*

Isolate	Patient age, y/sex	Collection date†	Genbank accession no.	Zika virus RT-PCR	Zika virus qRT-PCR C_t_	Viral load, copies/ mL	160-nt single-end metagenomic reads			250-nt paired-end Zika virus–specific enrichment		
							Genome recovery, %‡	Mean fold coverage		Genome recovery, %‡	Mean fold coverage	
Bahia01	72/F	2015 May 16	KX101066	Pos	34.6	1,042	23.1	0.4		65.3	16,288.2	
Bahia02	37/M	2015 May 5	KX101060	Pos	32.5	4,086	26.0	0.4		73.4	20,045.8	
Bahia03	35/M	2015 May 5	KX101061	Pos	32.8	3,272	1.1	0.0		77.7	220.0	
Bahia04	40/M	2015 Jun 1	KX101062	Pos	34.1	1,464	5.1	0.1		42.0	4,659.5	
Bahia05	U/M	2015 Dec 10	KX101063	Pos	33.7	1,901	5.0	0.1		42.8	8,547.5	
Bahia07	37/F	2015 Aug 29	KU940228	Pos	13.7	9.1 × 10^8^	100	3,603.5		ND	ND	
Bahia08	U/M	2015 Jul 15	KU940227	Pos	33.3	2,470	75.1	9.2		84.9	23,805.1	
Bahia09	40/F	2015 Apr 25	KU940224	Pos	29.9	23,121	99.98	41.5		ND	ND	
Bahia11	40/F	2015 Apr 27	KX101064	Pos	Neg (no C_t_)	NA	27.8	0.9		64.0	28,704.1	
Bahia12	36/M	2015 May 7	KX101067	Pos	34.2	1,327	11.2	0.2		50.4	10,461.8	
Bahia15	U/M	2016 Jan 25	KX101065	Pos	Neg (no C_t_)	NA	4.6	0.2		45.4	3,706.8	

*C_t_, cycle threshold; NA, not applicable; ND, not done; Neg, negative; Pos, positive; qRT-PCR, quantitative reverse transcription PCR; RT-PCR, reverse transcription PCR; U, unknown. †Samples were collected from Salvador in Bahia, Brazil, except for Bahia05, which was collected in Camaçari, Bahia, Brazil. ‡Assumes a genome size of 10,676 nt, the size of the prototype Brazilian Zika virus strain SPH2015 (KU321639).

Multiple sequence alignment was performed by using MAFFT version 7 (http://mafft.cbrc.jp/alignment/software/); maximum-likelihood (ML) and Bayesian phylogenetic inferences were determined by using PhyML version 3.0 (http://www.atgc-montpellier.fr/phyml/) and BEAST version 1.8.2 (http://beast.bio.ed.ac.uk/), respectively. The best-fit model was calculated by using jModelTest2 (https://github.com/ddarriba/jmodeltest2; details in Technical Appendix). Coding regions corresponding to the 11 complete or partial genomes from Bahia were aligned with all published and available near-complete Zika virus genomes and longer subgenomic regions (>1,500 nt) of the Asian genotype as of April 2016 (mean sequence size 8,402 nt with 1,652 distinct nucleotide site patterns). The ML phylogeny was reconstructed by using the best-fit general time-reversible nucleotide substitution model with a proportion of invariant sites (GTR+I). Statistical support for phylogenetic nodes was assessed by using a bootstrap approach with 1,000 bootstrap replicates. A Bayesian molecular clock phylogeny was estimated by using the best-fitting evolutionary model (*2*); specifically, a GTR+I substitution model with 3 components: a strict molecular clock, a Bayesian skyline coalescent prior, and a noninformative continuous time Markov chain reference prior for the molecular clock rate.

The isolates from patients in Salvador clustered together within 1 strongly supported clade (posterior probability 1.00, bootstrap support 100%, Bahia clade C) (Figure; Technical Appendix Figure 2). This support is notable; most Zika virus genomes in this clade are incomplete, and uncertainty is accounted for in phylogenetic inference. The tree topology accords with previous findings (*2*,*4*,^5^), and time to most recent common ancestor (TMRCA) of the epidemic in the Americas is similar to that previously estimated (*2*) (American epidemic clade A; Figure). The overall ML and molecular clock phylogenies exhibited many well-supported internal nodes with bootstrap support >60% and posterior probability >0.80 (Figure; Technical Appendix Figure 2), although several nodes near the ancestor of clade A were less well supported.

**Figure:**
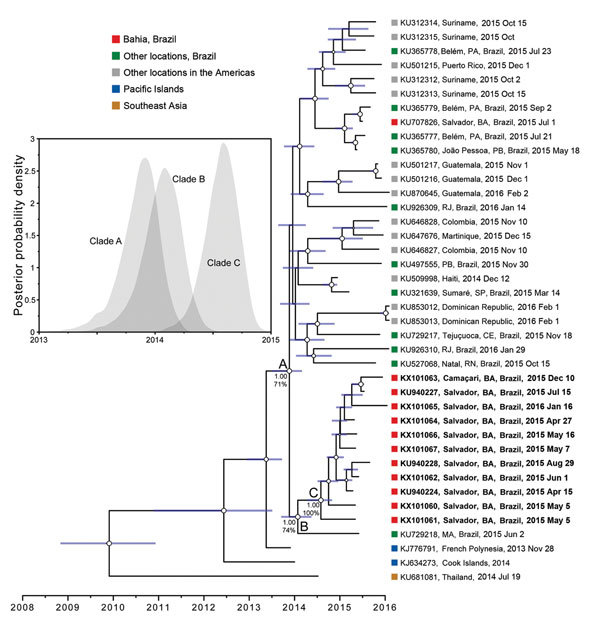
Timeframe of Zika virus outbreaks in the Americas. A molecular clock phylogeny is shown with the Zika virus outbreak lineage estimated from complete and partial (>1,500 nt) coding region sequences. For visual clarity, 5 basal Southeast Asia sequences (GenBank accession nos. HQ23499 [Malaysia, 1966]; EU545988 [Micronesia, 2007]; KU681082 [Philippines, 2012]; JN860885 [Cambodia, 2010]; and KU681081 [Thailand, 2013]) are not displayed. Blue horizontal bars represent 95% Bayesian credible intervals for divergence dates. A, B, and C denote the current American epidemic, the northeastern Brazil (Maranhão sequence and Bahia), and the Bahia clades, respectively; numbers next to the clade denote posterior probabilities and bootstrap scores in percentages. Circle sizes at each node represent the posterior probability support of that node. Taxa are labeled with the Genbank accession numbers, sampling location, and sampling date. Names of sequences generated in this study are in bold. The inset graph on the left shows the posterior probability distributions of the estimated ages (time to most recent common ancestor) for clades A, B, and C. The posterior probability density is plotted on the vertical axis as a function of time on the horizontal axis (tick marks designate 3-month intervals). Estimated ages were determined with BEAST version 1.8.2 (http://beast.bio.ed.ac.uk/) by using the best-fitting evolutionary model. The posterior probability distributions were visualized by using Tracer version 1.6 (http://tree.bio.ed.ac.uk/software/tracer/). Brazil states: BA, Bahia; CE, Ceará; MA, Maranhão; PA, Pará; PB, Paraíba; RN, Rio Grande do Norte; RJ, Rio de Janeiro; SP, São Paulo.

The updated phylogenetic analyses, including the newly identified clade C, suggest that Zika virus was introduced in Bahia during March–September 2014. An isolate from Maranhão in northeastern Brazil (≈1,000 km from Bahia) is ancestral to the Bahia clade (posterior probability 1.00, bootstrap support 74%, northeastern Brazil clade B) (Figure; Technical Appendix Figure 2). The TMRCA of clade B (comprising the Bahia clade and the Maranhão sequence) is estimated to be September 2013–April 2014, an early stage of the epidemic. This TMRCA is consistent with the hypothesis that Zika virus in the Americas originated in Brazil (*2*). A previously reported sequence from Bahia (*6*) clustered with an isolate from Belém in the state of Pará in northern Brazil, ≈3,000 km from Bahia (posterior probability 0.99, bootstrap support 81%) (Figure; Technical Appendix Figure 2). The patient denied history of travel, suggesting that multiple Zika virus lineages may circulate in Bahia.

## Conclusions

Our results suggest an early introduction and presence (mid-2014) of Zika virus in the Salvador region in Bahia, Brazil. Given the size of the cluster and statistical support for it, this lineage likely represents a large and sustained chain of transmission within Bahia state. Most cases of this Zika virus lineage clustered closely to a sequence from Maranhão, and we found evidence for an additional potential introduction to Bahia from Pará state. Consequently, Zika virus in Salvador during mid-2014 was likely introduced from other regions in Brazil rather than from outside the country. Current findings of Zika virus emergence in Bahia state during mid-2014 are consistent with first-trimester viral infection in pregnant women corresponding to the initial reported cases of fetal microcephaly, which began in January 2015 (*5*) and peaked in November 2015.

Broader sampling across Bahia is needed to determine whether the Salvador lineage (clade C) identified in this article comprises most Zika virus cases in the state. Brazil currently faces a major public health challenge from co-circulation of Zika, dengue, and chikungunya viruses (*2*–*4*,*14*,*15*). Additional molecular surveillance in the Americas and beyond is urgently needed to trace and predict transmission of Zika virus.

Technical Appendix Details of library construction and next-generation sequencing methods, phylogenetic analyses, and data availability for this study, including supplemental figures.
