# Supplementary material for: Distinct Zika Virus Lineage in Salvador, Bahia, Brazil
Source: Emerg Infect Dis. 2016 Oct;22(10):1788–92. doi: 10.3201/eid2210.160663 (PMC5038429; doi:10.3201/eid2210.160663)
Supplement: Technical Appendix — Details of library construction and next-generation sequencing methods, phylogenetic analyses, and data availability for this study, including supplemental figures. [file 16-0663-Techapp-s1.pdf]

Article DOI: http://dx.doi.org/10.3201/eid2210.160663

Distinct Zika Virus Lineage in Salvador, Bahia, Brazil

Technical Appendix

Next-Generation Sequencing Library Construction

RNA metagenomic libraries were constructed from the patients' serum sample as previously described (*1*,*2*). Total nucleic acid was extracted by using the QIAamp Viral RNA mini kit (Qiagen, Valencia, CA, USA). Extract (20 µL) was treated with Turbo DNase (Ambion, Waltham MA) and Baseline-ZERO DNase (Epicentre, Madison, WI, USA), followed by cleanup with the RNA Clean & Concentrator kit (Zymo Research, Irvine, CA, USA). Eluted RNA was reverse transcribed to cDNA by using Superscript III Reverse Transcriptase (Invitrogen, Carlsbad, CA, USA) mediated by random hexamers. Second strand synthesis was conducted by using Sequenase version 2.0 DNA Polymerase (Affymetrix, Santa Clara, CA, USA) for 10 minutes at 37°C following denaturation. Double-stranded cDNA was cleaned up by using the DNA Clean & Concentrator kit (Zymo Research, Irvine, CA, USA) and next-generation sequencing (NGS) libraries were generated from the entirety of the eluate by using the Nextera XT DNA Library Prep Kit (Illumina, San Diego, CA, USA). The Nextera XT library was cleaned by using Agencort AMPure XP beads (Beckman-Coulter, Brea, CA, USA). Dual-indexed, barcoded NGS libraries were quantitated on the Qubit 3.0 fluorometer (Fisher Scientific, Waltham, MA, USA), mixed equally by concentration, and quantified for size and concentration on the Bioanalyzer (Agilent, Santa Clara CA, USA) using the Agilent High Sensitivity dsDNA kit. Libraries were run on the Illumina HiSeq sequencing system (1 × 160 nt single-end runs and 2 × 250 nt paired-end runs). Metagenomic NGS data was analyzed for pathogens by using the automated Sequence-Based Ultra-Rapid Pathogen Identification (SURPI) computational pipeline (http://chiulab.ucsf.edu/surpi/) and the March 2015 National Center for Biotechnology Information nucleotide database (Bethesda, MD, USA) (*3*).

Phylogenetic Analyses

By using the MAFFT program (*4*), multiple sequence alignment of the coding regions corresponding to the 11 complete or partial genomes from Bahia, Brazil, which were recovered in this study were aligned together with all published and available near-complex Zika virus genomes and longer subgenomic regions (>1,500 nt) of the Asian genotype submitted to GenBank as of April 2016. Maximum likelihood and Bayesian phylogenetic inferences were determined by using PhyML version 3.0 (*5*; http://www.atgc-montpellier.fr/phyml/) and BEAST version 1.8.2 (*6*; http://beast.bio.ed.ac.uk/), respectively. We used the program jModelTest2 (*7*) to determine the best-fitted general time reversible nucleotide substitution model with a proportion of invariant sites (GTR+I).

Data Availability

The 11 assembled complete and partial Zika virus genomes recovered in this study have been deposited in GenBank (accession nos. KU940224, KU940227–KU940228, and KX101060–KX101067). Raw FASTQ sequence files with human reads removed by using Bowtie2 (*8*; http://bowtie-bio.sourceforge.net/bowtie2/index.shtml) in local alignment mode at default parameters have been deposited in the Sequence Read Archive (National Center for Biotechnology Information; accession no. SRP072069).

References

1. Greninger AL, Messacar K, Dunnebacke T, Naccache SN, Federman S, Bouquet J, et al. Clinical metagenomic identification of Balamuthia mandrillaris encephalitis and assembly of the draft genome: the continuing case for reference genome sequencing. Genome Med. 2015;7:113. http://dx.doi.org/10.1186/s13073-015-0235-2

2. Greninger AL, Naccache SN, Messacar K, Clayton A, Yu G, Somasekar S, et al. A novel outbreak enterovirus D68 strain associated with acute flaccid myelitis cases in the USA (2012–14): a retrospective cohort study. Lancet Infect Dis. 2015;15:671–82. http://dx.doi.org/10.1016/S1473-3099(15)70093-9

3. Naccache SN, Federman S, Veeraraghavan N, Zaharia M, Lee D, Samayoa E, et al. A cloud-compatible bioinformatics pipeline for ultrarapid pathogen identification from next-generation

sequencing of clinical samples. Genome Res. 2014;24:1180–92. http://dx.doi.org/10.1101/gr.171934.113

4. Katoh K, Asimenos G, Toh H. Multiple alignment of DNA sequences with MAFFT. Methods Mol Biol. 2009;537:39–64. http://dx.doi.org/10.1007/978-1-59745-251-9_3

5. Guindon S, Dufayard JF, Lefort V, Anisimova M, Hordijk W, Gascuel O. New algorithms and methods to estimate maximum-likelihood phylogenies: assessing the performance of PhyML 3.0. Syst Biol. 2010;59:307–21. http://dx.doi.org/10.1093/sysbio/syq010

6. Drummond AJ, Suchard MA, Xie D, Rambaut A. Bayesian phylogenetics with BEAUti and the BEAST 1.7. Mol Biol Evol. 2012;29:1969–73. http://dx.doi.org/10.1093/molbev/mss075

7. Darriba D, Taboada GL, Doallo R, Posada D. jModelTest 2: more models, new heuristics and parallel computing. Nat Methods. 2012;9:772. http://dx.doi.org/10.1038/nmeth.2109

8. Langmead B, Salzberg SL. Fast gapped-read alignment with Bowtie 2. Nat Methods. 2012;9:357–9. http://dx.doi.org/10.1038/nmeth.1923

9. Edgar RC. MUSCLE: multiple sequence alignment with high accuracy and high throughput. Nucleic Acids Res. 2004;32:1792–7. http://dx.doi.org/10.1093/nar/gkh340

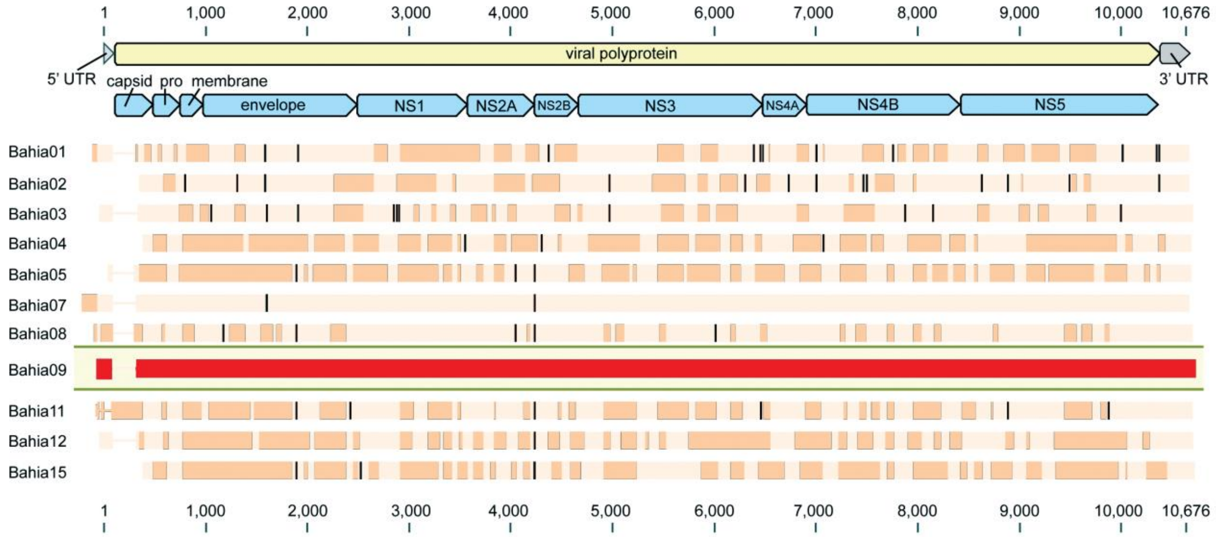

Technical Appendix Figure 1. Multiple sequence alignment of 11 whole and partial Zika virus genomes from the Salvador region of Bahia, Brazil. Viral genomes were aligned by using the MUSCLE program (*9*; http://www.drive5.com/muscle/) at default parameters. Aligned genomic regions are shown in light tan, with missing genomic regions from partially recovered genomes shaded in dark tan. Single nucleotide variants differing from the fully sequenced Bahia09 strain (in red) are plotted as vertical black lines according to nucleotide position along the viral genome (horizontal axis). The single nucleotide variant patterns differ for each strain; none are identical to Bahia07 or Bahia09, the Zika virus isolates with the highest viral titers. NS, nonstructural gene; UTR, untranslated region.

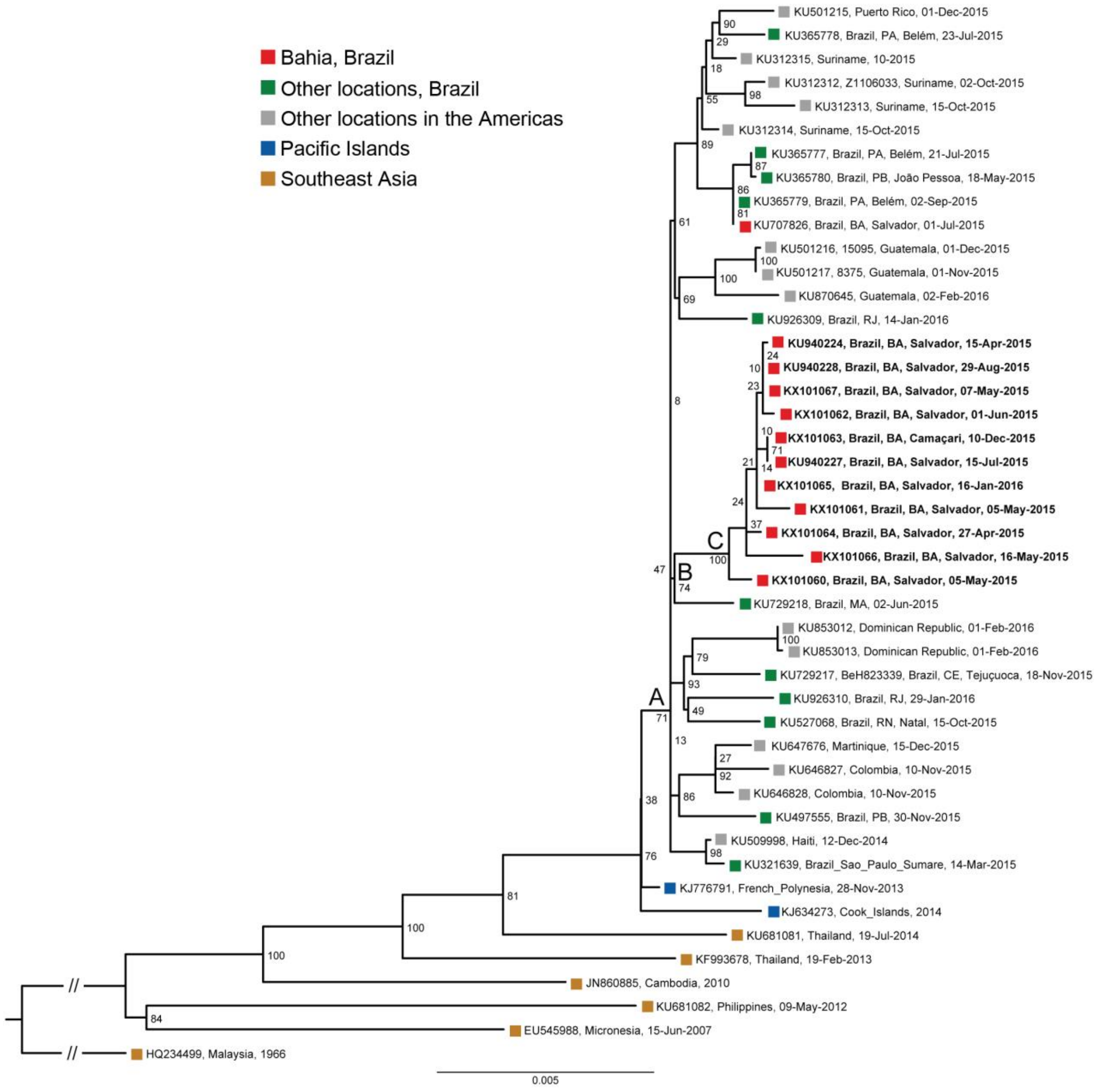

Technical Appendix Figure 2. Maximum likelihood phylogeny of Zika virus Asian genotype. The tree was estimated from complete and partial (>1,500 nt) coding region sequences. Taxa are labeled with GenBank accession numbers (http://www.ncbi.nlm.nih.gov/genbank/), sampling locations, and sampling dates. Names of sequences generated in this study are shown in bold. Branch lengths are drawn proportionally to the number of nucleotide substitutions per position (indicated by the scale bar). Numbers next to phylogenetic nodes denote bootstrap percentages (1,000 replicates). Brazil states: BA, Bahia; CE, Ceará; MA, Maranhão; PA, Pará; PB, Paraíba; SP, São Paulo; RN, Rio Grande do Norte; RJ, Rio de Janeiro.
